# OLDER BLACK LESBIANS' NEEDS AND EXPECTATIONS IN RELATION TO LONG-TERM CARE FACILITY USE

**DOI:** 10.1093/geroni/igac059.197

**Published:** 2022-12-20

**Authors:** Meki Singleton, Mary Anne Adams, Tonia Poteat

**Affiliations:** University of Southern California, Los Angeles, California, United States; ZAMI NOBLA: National Organization of Black Lesbians on Aging, Atlanta, Georgia, United States; University of North Carolina Chapel HIll, Chapel Hill, North Carolina, United States

## Abstract

The older adult sexual minority (SM) population encompasses a vast array of individuals from diverse backgrounds. However, there is a dearth of gerontological research that focuses on the experiences of SM subgroups, particularly older Black lesbians. The purpose of this study was to explore older Black lesbians’ needs and expectations in relation to the utilization of long-term care (LTC) facilities. We conducted secondary data analysis using data from 14 focus groups (n=100) that discussed health and aging with older Black lesbians. Transcriptions were analyzed in NVivo 12 using content analysis and structural coding. Three major themes were identified in relation to needs and expectations for LTC facility use: (1) consideration of or plans established to utilize a LTC facility, (2) concern for care facility environment, and (3) a desire to build one’s own community instead of LTC use. Within these themes, prominent topics included having to rely on LTC due to a lack of family or social support, the possibility of being isolated and stifling their lesbian identity and creating communities of mutual support to avoid facility-based care. These findings illustrate how older Black lesbians are planning for a potential need for LTC, their concerns about utilizing LTC, and alternative approaches to avoid LTC use. There remains a continued need for LTC communities that are inclusive and supportive of SM older adults as well as more SM-only communities where older adults can live openly and authentically.

